# Assessment of plasma *p*‐tau217 performance: diagnostic accuracy and pathology discrimination

**DOI:** 10.1002/alz70856_100263

**Published:** 2025-12-25

**Authors:** Farida Dakterzada, Ricard López Ortega, Nuria Tahan, Iolanda Riba Llena, Maria Ruiz Julián, Alfonso Arias, Gerard Piñol‐Ripoll

**Affiliations:** ^1^ Hospital Universitari Santa Maria de Lleida, IRBLleida, Lleida, Spain; ^2^ Hospital Universitari Arnau de Vilanova, Lleida, Catalonia, Spain; ^3^ Hospital Universitari Santa Maria Lleida, Cognitive Impairment Unit, Lleida, Spain, Lleida, Spain; ^4^ Institut de Recerca Biomédica de Lleida (IRBLLeida), Lleida, Spain; ^5^ Hospital Universitari Santa Maria, LLeida, Spain; ^6^ Hospital Universitari Santa Maria LLeida, LLeida, Spain; ^7^ Cognitive Disorder Unit, Hospital Universitari Santa Maria, Lleida, Spain

## Abstract

**Background:**

Our objectives were to determine the diagnostic accuracy of plasma *p*‐tau217 and its discriminative power for the Alzheimer's disease‐related pathology, and to determine the cut‐off that optimize the concordance with Aβ42/Aβ40 positive vs negative status.

**Method:**

127 patients with AD (*n* = 30), MCI (*n* = 81), and non‐AD dementias (*n* = 16) were included. Aβ42, Aβ40, total tau, and phosphorylated tau181 (*p*‐tau181) were quantified in CSF and Aβ42, Aβ40, *p*‐tau181, and *p*‐tau217 were measured in plasma using the Lumipulse platform (Fujireibio).

**Result:**

We found a significant correlation between plasma levels of *p*‐tau217 and CSF levels of *p*‐tau181 (*r* = 0.539), Aβ42 (*r* = −0.443), and Aβ42/Aβ40 (*r* = −0.594). Regarding diagnostic accuracy, the discriminatory power of *p*‐tau217 in separating AD from non‐AD dementia patients (AUC 0.856, 95% CI 0.715‐0.996) was comparable with CSF Aβ42/Aβ40 (AUC 0.879, 95% CI 0.592‐0.887) and significantly different from plasma *p*‐tau181 (AUC 0.662, 95% CI 0.500‐0.823). In addition, the plasma levels of *p*‐tau217 were associated with the brain AD‐related pathology quantified in CSF. Finally, plasma *p*‐tau217 (AUC 0.919, 95% CI 0.857‐0.981) showed a high consistency with amyloid pathology (CSF Aβ42/40) at ≥ 0.234 pg/mL cut‐off. The two cut‐offs approach (> 0.314 pg/mL positive and < 0.161 pg/mL negative for *p*‐tau217) revealed that nearly 84.7% of the patients can be diagnosed as negative or positive for *p*‐tau217 with a sensitivity and specificity of 94.7% and 93.9%, respectively.

**Conclusion:**

*p*‐tau217 blood biomarker has a high diagnostic accuracy and, therefore, it can be a useful tool for screening of those patients that will need a lumbar puncture for AD diagnosis.

